# Dynamic Susceptibility Contrast Magnetic Resonance Imaging Protocol of the Normal Canine Brain

**DOI:** 10.3389/fvets.2017.00041

**Published:** 2017-03-21

**Authors:** Krystina L. Stadler, Anthony P. Pease, Elizabeth A. Ballegeer

**Affiliations:** ^1^Department of Small Animal Clinical Sciences, Michigan State University College of Veterinary Medicine, East Lansing, MI, USA

**Keywords:** canine, perfusion, dynamic susceptibility contrast, magnetic resonance imaging, neuroimaging

## Abstract

Perfusion magnetic resonance imaging (MRI), specifically dynamic susceptibility MRI (DSC-MRI) is routinely performed as a supplement to conventional MRI in human medicine for patients with intracranial neoplasia and cerebrovascular events. There is minimal data on the use of DSC-MRI in veterinary patients and a DSC-MRI protocol in the veterinary patient has not been described. Sixteen normal dogs, 6 years or older were recruited for this study. The sample population included 11 large dogs (>11 kg) and 5 small dogs (<11 kg). DSC-MRI was performed on a 1.5-T MRI using an adjusted protocol inherent to the MRI. Contrast media was injected using an automatic power injector. Injections were made after five MR measurements were obtained. Following image acquisition, an arterial input function (AIF) graph mapping the transit time of contrast within the cerebral arteries was generated. The manually selected time points along this graph were used to compute perfusion maps. A dose and rate of 0.1 mmol/kg gadolinium-based contrast media at 3 ml/s followed by 10 ml saline flush at 3 ml/s was used in all dogs greater than 11 kg. In all dogs >11 kg, a useable AIF and perfusion map was generated. One dog less than 11 kg received the same contrast dose and rate. In this patient, the protocol did not generate a useable AIF. The remainder of the dogs less than 11 kg followed a protocol of 0.2 mmol/kg gadolinium-based contrast media at 1.5 ml/s with a 10 ml saline flush at 1.5 ml/s. A useable AIF and perfusion map was generated in the remaining dogs <11 kg using the higher contrast dose and slower rate protocol. This study establishes a contrast dose and administration rate for canine DSC-MRI imaging that is different in dogs greater than 11 kg compared to dogs less than 11 kg. These protocols may be used for future applications to evaluate hemodynamic disturbances in canine intracranial pathology.

## Introduction

Perfusion magnetic resonance imaging (MRI) is an important non-invasive tool in human medicine for evaluating cerebral hemodynamics (1). Two contrast-based perfusion imaging sequences are described: dynamic susceptibility weighted dynamic susceptibility MRI (DSC-MRI) and dynamic contrast-enhanced MRI (DCE-MRI) (2). In human medicine, DSC-MRI is the most widely used method to measure brain perfusion due to the software availability and ease of use (2, 3).

Dynamic susceptibility MRI images the first pass of a bolus of gadolinium-based contrast through the brain by a series of T2*-weighted (T2*W) MRI images to generate a signal intensity to time curve, also known as an arterial input function (AIF). The susceptibility of the contrast causes a decrease in signal intensity, which leads to a signal loss in the AIF. From this curve, multiple hemodynamic parameters such as time to peak, mean transit time (MTT), cerebral blood flow (CBF), and cerebral blood volume (CBV) can be determined for each pixel and perfusion maps are generated (2, 4). A major assumption in DSC-MRI studies is that contrast remains within the blood vessels such that a susceptibility gradient can be induced between the intravascular and extravascular space, this assumption can lead to underestimation of perfusion, specifically in brain tumors (5, 6). DCE-MRI allows for better quantitative measurement of the blood–brain barrier, assessing the tissue permeability and the extracellular space, the values are sensitive to tumor growth and treatment response (7). DCE-MRI techniques involve serial T1-weighted (T1W) images before, during, and after gadolinium contrast administration (7). The reason DSC-MRI is used more often in the clinical setting is due to the complexity in image acquisition and post-processing of DCE-MRI data and the lack of widely available software (2). In contrast, most commercially available MRI scanners have inherent acquisition parameters and software for DSC-MRI. To the authors’ knowledge, the only descriptive study in veterinary medicine using perfusion MRI is a quantitative perfusion study using DCE-MRI in dogs with intracranial neoplasia using 3-T MRI and manual contrast injection (8). Since both DSC-MRI and DCE-MRI involve dynamic imaging acquisitions, the use of an automated power injector is considered necessary to allow a fast injection needed for DSC-MRI and reproducible administration DCE-MRI perfusion (2). No studies using DSC-MRI in the normal canine brain have been described.

Extensive research and clinical applications of DSC-MRI are described in human patients with stroke (9, 10), neoplasia (11–13), dementia (14), anesthesia (15), epilepsy (16), and trauma (17, 18). Of the described clinical applications of DSC-MRI in human medicine, the use of DSC-MRI in intracranial neoplasia grading and therapeutic monitoring is the most clinically relevant to our canine patients and translational research (12, 19–24). Despite the amount of data available in human medicine, few reports in veterinary medicine have been published, with most available studies using animals as a model of disease. Examples include canine studies of ischemic stroke (25) and brain changes secondary to cardiac arrest (26). Within the veterinary literature, to the authors’ knowledge, in addition to the previously mentioned DCE-MRI study, two descriptive reviews are available for vascular and perfusion imaging in the canine brain, with no protocol details (27, 28).

A protocol for DSC-MRI at 1.5 T in the normal canine brain with a power injector has not been described. The aim of this study was to determine a DSC-MRI protocol for the normal canine brain.

## Methods

The study protocol was designed in accordance with and approved by the Michigan State University animal care and use committee. The study was a prospective cohort study performed by recruiting client owned, consented healthy dogs, middle to senior in age (≥6 years).

All study dogs underwent a physical exam and bloodwork (complete blood count and serum chemistry) to ensure the animals were healthy and able to undergo anesthesia. All dogs were imaged under general anesthesia. All dogs received butorphanol (0.2 mg/kg, IV, or IM) as a premedication prior to induction. Varying by case, acepromazine (0.05 mg/kg, IV, or IM) was also administered as a premedication. General anesthesia was induced with propofol (4 mg/kg, IV, titrated to effect). The dogs were intubated and maintained on light anesthetic plane using sevofluorane gas anesthesia. Depending on patient size, an 18- or 20-gauge catheter was placed within the cephalic vein approximately 20 min after premedication administration and prior to anesthetic induction for anesthetic intravenous fluid delivery and contrast bolus administration.

Images were acquired using a 1.5-T Siemens Espree (Melvin, PA, USA) and an 8-channel coil head or knee coil. In general, large dogs were placed in the head coil due to its greater internal diameter and smaller dogs in the knee coil to minimize air gap between the coil and patient. All dogs had an abbreviated pre-contrast conventional brain MRI study including transverse T1W and fluid attenuating inversion recovery sequences.

Dynamic susceptibility MRI images were acquired using a first pass gadolinium contrast-enhanced T2*W echo-planar image sequence (Siemens, ep2d_perf) with 50 measurements. Each measurement ranged from 1.5 to 2 s long depending on number of slices. Continuous transverse slices throughout the brain were made with each measurement. The number of slices was dependent on patient size. The image acquisition parameters were as follows repetition time: average 2,008 ms (range: 1,690–2,250), echo time: 62.4 ms, flip angle: 90°, slice thickness: 4 mm, field of view: 140 or 150, number of excitations: 1, and matrix size of 64 × 64 × 16. Paramagnetic contrast media, gadobenate dimeglumine (Multihance^®^), was injected into the cephalic vein catheter using an automatic power injector (Spectris Solaris^®^, Medrad) after five measurements. All dogs greater than 11 kg received a 0.1 mmol/kg contrast bolus at 3 ml/s followed by10 ml saline flush at 3 ml/s. One dog less than 11 kg received the same dose and rate. The remainder of dogs less than 11 kg followed a modified protocol of 0.2 mmol/kg contrast at 1.5 ml/s with a 10 ml saline flush at 1.5 ml/s. After perfusion data was obtained, all dogs underwent a post-contrast transverse T1W study.

**Figure 1:**
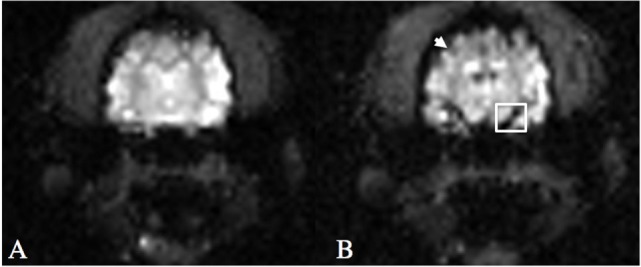
T2*W echo-planar imaging sequence following gadolinium-based contrast injection at T = 0 (A) and time of peak arterial contrast susceptibility (B) at the level of the middle cerebral artery. Note the hypointense cortical arteries around the periphery of the cerebrum (arrow) and the middle cerebral artery (box) at peak contrast susceptibility.

**Figure 2:**
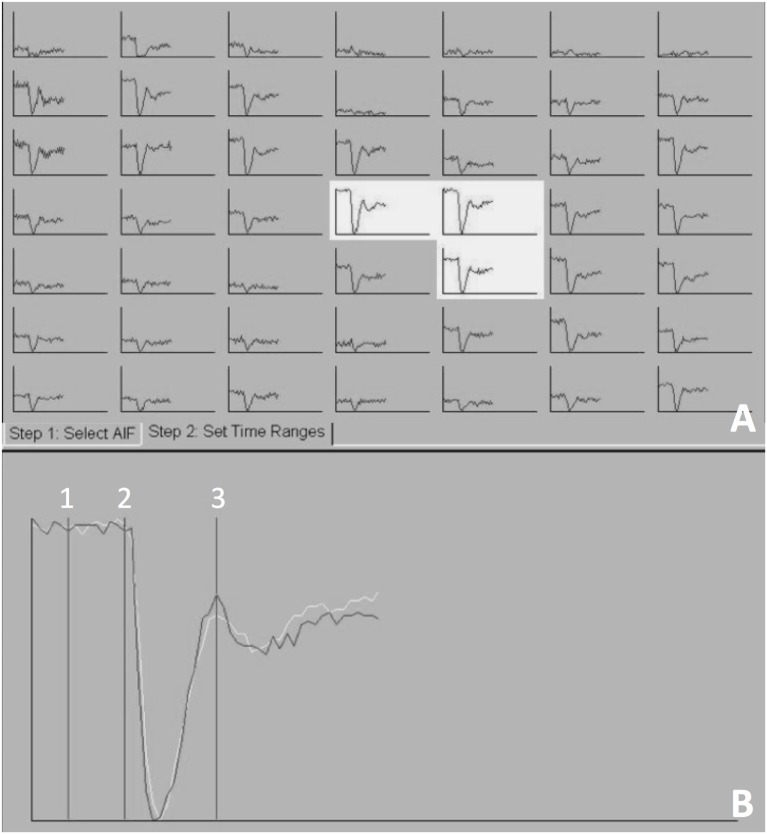
The arterial input function (AIF) generated at the level of the middle cerebral artery, mapping T2*signal (*y*-axis) against time (*x*-axis). Three AIF graphs were selected **(A)** and a representative averaged AIF was generated **(B)**. Three points on the averaged AIF were selected 1—the baseline prior to the contrast arrival, 2—point on the graph immediately prior to signal loss peak, and 3—time immediately after return to baseline.

**Figure 3:**
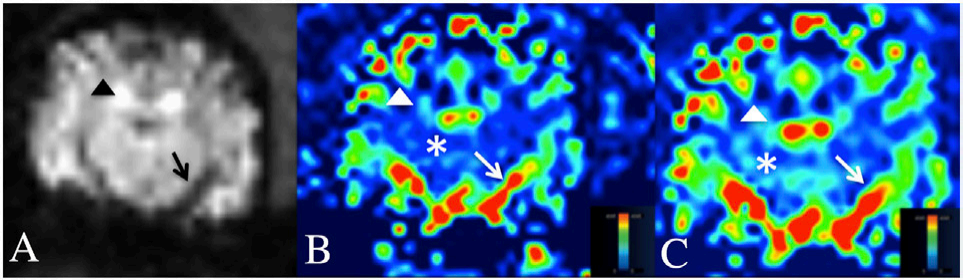
**Dynamic susceptibility MRI (DSC-MRI) in the normal canine brain**. **(A)** DSC-MRI T2*W image at the level of the middle cerebral artery. Note the hypointensity of the cortical arteries (black arrow head) and middle cerebral artery (black arrow) during the arterial first pass of the gadolinium contrast bolus. **(B,C)** Cerebral blood volume **(B)** and cerebral blood flow **(C)** maps at the level of the middle cerebral artery. A blue-red scale is used on these maps, where red is high perfusion and blue is low perfusion. Note the red middle cerebral arteries and red–green cortical arteries (white arrow and arrow head) and the blue–green cerebral parenchyma (white asterisks).

Post-processing of DSC-MRI images was performed using the Siemens MRI analysis software (syngo.MR.NeuroPerfusion^®^) by one of the authors (Krystina L. Stadler). This commercially available software generates multiple AIF graphs based on a defined region of interest (ROI) centered over an area of high perfusion. For this study, the middle cerebral artery was used as the ROI (Figure 1). The AIF graphs T2*signal strength (*y*-axis) against time (*x*-axis). As the bolus arrives to the ROI, there is a drop-in signal strength (susceptibility on T2*W images), which remains until redistribution occurs (Figure 2). For further analysis, three of the AIF graphs were selected (Figure 2A). The selection of the three best AIF was subjective, defined by the AIF with the greatest defined peak and least amount of background noise. The graphs are then averaged by the software and used to generative a representative averaged AIF that is used to create cerebral perfusion maps (Figure 2B). From the generated average, AIF, the baseline prior to the contrast arrival, point on the graph immediately prior to signal loss peak, and time immediately after return to baseline were selected (Figure 2B). After these points are selected, the software generates relative color perfusion maps of the entire brain based on the perfusion imaging physics in which CBV is represented by the integrated area under the AIF curve, relative MTT is the width of the curve and CBF as the ratio between CBV and MTT. The software uses a blue–green–red color scheme for CBV and CBF maps with red being highly perfused, dark blue indicating extremely low perfusion, and green being intermediate perfusion (Figure 3).

## Results

Sixteen dogs met the inclusion criteria for this study. Multiple breeds were represented including: Staffordshire terrier (*n* = 4), mixed breed dogs (3), Laborador retriever (1 pure, one crossbreed), and one of each the following: Papillion, Beagle, Shiba Inu, Jack Russel Terrier, German Shepherd and Chihuahua. Nine dogs were spayed females, five were neutered males, and one was an intact male. The mean dog weight was 21.2 kg (range: 3.7–40.4 kg). Body condition score was not evaluated.

The injection protocol of 0.1 mmol/kg gadolinium followed by 10 ml saline flush at an injection rate of 3 ml/s generated a useable perfusion map in all dogs >11 kg (*n* = 11). In a small dog, weighing 10.5 kg, this protocol did not generate a useable map. This dog had evidence of contrast administration noted on the post-contrast T1W images. Following this dog, the protocol was augmented and a useable perfusion map was generated in the remaining dogs weighing less than 11 kg (*n* = 4) receiving the 0.2 mmol/kg of contrast medium and 10 ml saline flush at an injection rate of 1.5 ml/s protocol.

On the CBV and CBF maps, the large arterial structures (cerebral and cortical arteries) of the cerebrum were red on perfusion maps (high perfusion) with the smaller peripheral branches of these structures being green. The normal cerebral parenchyma was predominately royal blue (low perfusion) with small foci of light blue (low, but slightly higher perfusion). Areas of no perfusion, such as the lateral ventricles were dark blue.

## Discussion

The main clinical indications for perfusion imaging are neoplasia or a cerebrovascular event. Therefore, dogs 6 years or older were selected for this study to best match the age of dogs more commonly affected with intracranial diseases diagnosed by DSC-MRI. In human medicine, quantitative MRI CBF changes with age; however, in adults, qualitative maps are generated without changing protocol (29). This study did not validate this protocol in dogs younger than 6 years. The cause of the 0.1 mmol/kg at 3 ml/s protocol failure in one of the small dogs is unknown. This dog was the first small dog to be included in the study and was imaged after multiple successful DSC-MRI in dogs larger than this one. The augmentation of the protocol for smaller dogs was based on protocols successfully used in neonatal humans and piglets (1, 30, 31). Ideally, additional small dogs would have been tested at the protocol to see if the failure occurred repeatedly, however, given the funding available and limited availability of small dogs fitting our inclusion criteria, this was not possible and is a limitation to this study. The dog that failed to generate perfusion maps was 10.5 kg and thus the threshold weight limit between small and large dogs was placed at 11 kg. The closest large dog weight to the 11 kg cutoff was 15 kg; this dog generated a useable AIF with the lower contrast dose, higher rate protocol. The authors postulate that the small volume of contrast and/or the rate of contrast administration are a contributing factor of the lower contrast/higher rate DSC-MRI protocol not working in the small dogs. In the authors’ clinical experience, the lower contrast dose/higher rate was not successful in additional small dogs; these dogs were not included in this study because they had a known intracranial disease, which may have resultant alterations in their cerebrovascular perfusion. Dosing by mmol/body weight (kg) is convention at our institution. Body condition score was not factored into contrast dosing. To the author’s knowledge, the relationship between patient body condition and gadolinium-based contrast media dosage has not been described. It is unknown if altering the DSC-MRI protocol for ideal body condition weight in patients that are over or under conditioned into the small or large dog protocol would affect the AIF and is a limitation of this study.

In this study, the middle cerebral artery was chosen to generate the AIF because of its reproducible susceptibility that occurred on arterial first pass and for continuity in map generation. Although not assessed in this study, the placement of the ROI on any large artery, preferably in the same slice as your pathology is recommended in human medicine and should generate a similar perfusion map (32). This study’s aim was to describe a contrast protocol that can be used in the canine patients for future studies evaluating cerebral perfusion. The testing of multiple different contrast doses and bolus rates were beyond the scope of this study. Additional studies with a large population of normal dogs may help to determine if any alterations to the recommended dose and rate could be implemented. Gadolinium is a considered a safe contrast medium; doubling the dose is common in pediatric human medicine (1). Thus, the current recommended dose for small dogs should have minimal ill effect to the patient and provide consistent diagnostic DSC-MRI perfusion maps.

It is the authors’ belief that the addition of perfusion maps to conventional MRI images in canine patients will optimize the diagnostic accuracy of intracranial lesions and aid in treatment and prognosis as it has in human medicine. This is the study in veterinary medicine to describe a protocol for the use of DSC-MRI in canine patients. This study used a 1.5-T MRI and a power injector to obtain repeatable, uniform perfusion maps. This study establishes a protocol for canine 1.5-T DSC-MRI imaging that is different in large and small dogs. These protocols may be used for future applications to evaluate hemodynamic disturbances in canine intracranial pathology.

## Author Contributions

KS: principal investigator and primary author of the manuscript. As PI, KS was responsible for research development and adjustment of the methods, grantsmanship, and authoring of the manuscript. This research was done as part of KS’s radiology residency. AP and EB: both faculty resident and research mentors responsible for editing and approving methodology prior to initiation, editing, and co-PI on grants and IACUC, as well as internal editing of the manuscript.

## Conflict of Interest Statement

The authors declare that the research was conducted in the absence of any commercial or financial relationships that could be construed as a potential conflict of interest.

## Abbreviations

AIF, arterial input function; CBF, cerebral blood flow; CBV, cerebral blood volume; DCE-MRI, dynamic contrast-enhanced magnetic resonance imaging; DSC-MRI, dynamic susceptibility contrast magnetic resonance imaging; FLAIR, fluid attenuating inversion recovery; MRI, magnetic resonance imaging; MTT, mean transit time; ROI, region of interest; T1W, T1-weighted; T2(*)W, T2(*)-weighted.
